# Long-term efficacy of a rural community-based integrated intervention for prevention and management of chronic obstructive pulmonary disease: a cluster randomized controlled trial in China's rural areas

**DOI:** 10.1590/1414-431X20154385

**Published:** 2015-08-28

**Authors:** X. Yuan, Y. Tao, J.P. Zhao, X.S. Liu, W.N. Xiong, J.G. Xie, W. Ni, Y.J. Xu, H.G. Liu

**Affiliations:** 1 Department of Respiratory and Critical Care Medicine, Tongji Hospital, Huazhong University of Science and Technology, Wuhan, China; 2 Department of Respiratory and Critical Care Medicine, Xinhua Hospital of Hubei Province, Wuhan, China; 3 Department of Respiratory and Critical Care Medicine, Nanjing First Hospital, Nanjing Medical University, Nanjing, China

**Keywords:** Chronic obstructive pulmonary disease, Randomized controlled trial, Integrated intervention, Rural area, Lung function, Smoking cessation

## Abstract

This study aimed to assess the efficacy of a rural community-based integrated intervention for early prevention and management of chronic obstructive pulmonary disease (COPD) in China. This 18-year cluster-randomized controlled trial encompassing 15 villages included 1008 patients (454 men and 40 women in the intervention group [mean age, 54 ± 10 years]; 482 men and 32 women in the control group [mean age, 53 ± 10 years]) with confirmed COPD or at risk for COPD. Villages were randomly assigned to the intervention or the control group, and study participants residing within the villages received treatment accordingly. Intervention group patients took part in a program that included systematic health education, smoking cessation counseling, and education on management of COPD. Control group patients received usual care. The groups were compared after 18 years regarding the incidence of COPD, decline in lung function, and mortality of COPD. COPD incidence was lower in the intervention group than in the control group (10% *vs* 16%, <0.05). A decline in lung function was also significantly delayed in the intervention group compared to the control group of COPD and high-risk patients. The intervention group showed significant improvement in smoking cessation compared with the control group, and smokers in the intervention group had lower smoking indices than in the control group (350 *vs* 450, <0.05). The intervention group also had a significantly lower cumulative COPD-related death rate than the control group (37% *vs* 47%, <0.05). A rural community-based integrated intervention is effective in reducing the incidence of COPD among those at risk, delaying a decline in lung function in COPD patients and those at risk, and reducing mortality of COPD.

## Introduction

Chronic obstructive pulmonary disease (COPD) is characterized by a limitation in airflow that is not fully reversible. Currently, COPD is widespread throughout the world (1). The overall prevalence of COPD in China is 8.2% and this prevalence is significantly higher in rural areas compared with urban areas (2). COPD has become a major worldwide public health problem because of its already high and steadily increasing prevalence.

Fortunately, COPD is a preventable and treatable disease. Quitting smoking, nutritional support, and pulmonary rehabilitation are effective measures for prevention and treatment of COPD (3–6). COPD is a long-term, chronic disease. Currently, patients with COPD receive treatment as outpatients and, therefore, spend much of their time in their communities rather than in hospitals. Recent studies that examined two useful community-based comprehensive interventions for COPD have produced encouraging results (7,8).

Currently, interventions for COPD are mainly implemented in cities or economically developed areas, while little attention has been paid to economically underdeveloped or rural areas, especially in China. Because aspects of local social environments, economic conditions, and cultures all affect development of COPD and its outcomes, findings from studies that are conducted in urban or affluent areas may not be applicable in other areas.

The primary aim of this study was to investigate the effects of an integrated intervention for prevention of COPD. The secondary aim was to investigate the differences in decline in lung function between the intervention and control groups. Therefore, an 18-year rural community-based comprehensive intervention study was carried out to evaluate the efficacy of an integrated intervention for early prevention and management of COPD.

## Material and Methods

### Selection of areas and populations

This rural community-based cluster-randomized controlled trial was conducted from May 1992 to September 2010 in some villages in Haokou Township, Qianjiang, China. This area was chosen because it is typical of China's rural areas, containing no modern industry in the immediate or surrounding areas. People in these areas mainly rely on agriculture and handicrafts to make a living, and spend most of their time inside their homes. Another important reason for choosing this area was that there are complete village clinics in every village or pair of neighboring villages, with permanently stationed health care personnel and a fixed treatment room in each clinic. These local health personnel (called “village doctors” in China) are always the original owners of the clinics, and are well-educated. They have received specialist medical training and have passed a medical licensing examination. They always have close connections with the local residents, and this helps to improve their patients' adherence to treatment protocols.

**Figure 1:**
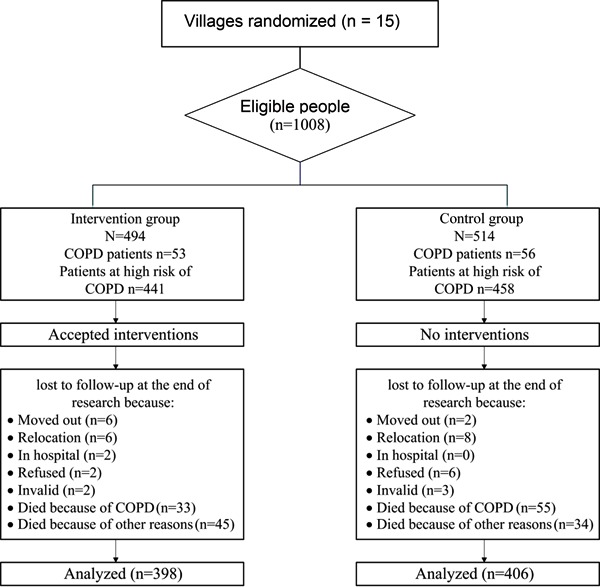
Flow chart of participants in the study.

Computer-generated random selection was used to select 15 villages from the 48 villages in Haokou Town. Villages were used as the study units. Each village was assigned to the intervention or control group by simple randomization (a coin toss). Among 16,511 individuals over 18 years of age living in these villages, 1,062 over 40 years of age met the diagnostic criteria for COPD or were found to have a high risk of COPD by physical examination and spirometry testing. Fifty-four individuals were excluded from the analyses for various reasons, including incomplete information such as census data or moving to another area after the study began. Finally, a total of 1,008 individuals over 40 years of age living in the 15 selected villages were found to fulfill criteria for inclusion in the integrated intervention study. Follow-up interviews were conducted with these subjects every year over the next 18 years, until 2010 (Figure 1).

### Spirometry tests

All of the participants underwent standard spirometry tests. The tests were conducted by technicians from the authors' hospital who had received rigorous training and passed the necessary examination. In summary, the patients first inhaled 400 µg of albuterol sulfate aerosol over a period of 10-20 min and were then examined. The diagnostic criterion for COPD was a post-bronchodilator forced expiratory volume in 1 s (FEV_1_)/forced vital capacity (FVC) ratio under 0.7. The levels of severity of the subjects' COPD were classified as follows: mild (FEV_1_≥80% of the predicted value); moderate (FEV_1_ <80% and ≥50% of the predicted value); and severe (FEV_1_<50% and ≥30% of the predicted value). Very severe patients (FEV_1_ <30% of the predicted value or FEV_1_<50% and ≥30% of the predicted value and the presence of chronic respiratory failure) were not included in the study. Patients who did not meet the diagnostic criteria (i.e., had an FEV_1_/FVC ≥0.7) but had a smoking history and were currently smoking, or were suffering from second-hand smoke, chronic cough, with increased sputum in the morning and other symptoms, were considered to be at high risk of developing COPD.

All of the COPD participants were in a stable phase of the disease and did not receive any drugs, such as bronchodilators when they entered the study. Patients were excluded if they had diseases such as asthma, bronchiectasis, interstitial lung disease, active tuberculosis, chronic heart failure, cancer, or other diseases that could potentially affect the spirometry test. Patients were also excluded if they could not communicate well with the respiratory specialist or who were unwilling to participate in the study. Each participant either signed an informed consent form or had one signed by a member of his/her immediate family. This study was carried out in accordance with the requirements of the Declaration of Helsinki. Ethics approval was obtained from the local Health Department and the research ethics board of Tongji Medical College.

### Comprehensive intervention

The patients' villages were randomly divided into control and experimental intervention groups, with each group of villages containing patients who were diagnosed with varying levels of severity of COPD, as well as the population judged to be at high risk for COPD. Participants in the experimental intervention group received a comprehensive intervention. The intervention consisted of an intensive intervention phase and an active maintenance phase. In the intensive intervention phase, a respiratory specialist from the authors' hospital performed specific interventions for 1 month or more, at 6-month intervals. In the active maintenance phase, the interventions were mainly performed by local health personnel who received regular supervision from a respiratory specialist and several public health experts from the authors' hospital.

In the first step of the comprehensive intervention, participants were provided with basic knowledge through classroom teaching, because the local residents generally followed an “out at sunrise, in at sunset” lifestyle, had received only a limited education, and had difficulties with understanding and grasping new ideas. Every effort was made to offer them clear and simple information on general health issues, smoking cessation, COPD management, and pulmonary rehabilitation (Table 1).

**Table:**
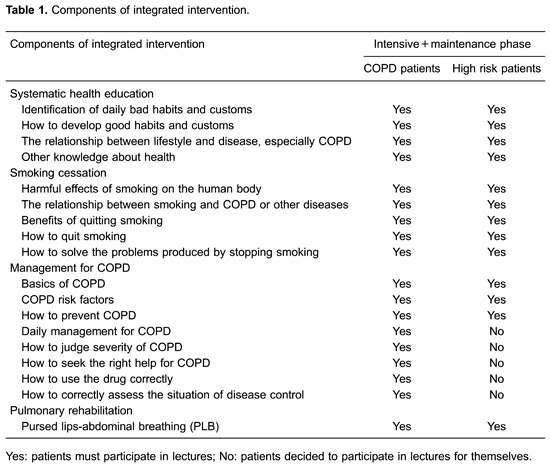

The COPD patients in the control villages were treated with conventional therapy in accordance with guidelines. Neither they nor those considered to be at high risk for COPD received additional medical aid in the control villages.

### Follow-up and measurement of health outcomes

Because of the large number of participants and funding issues, pulmonary function testing was not performed every year for all of the patients. However, if participants who were considered to be at high risk of COPD had respiratory symptoms, such as coughing or wheezing that suggested that they might have progressed to COPD, spirometry tests were performed to confirm their condition. For patients who were already suffering from COPD, spirometry tests were also carried out to facilitate better treatment when their symptoms became aggravated. All of the participants had spirometry tests at the end of the trial.

The cumulative case fatality rates for COPD in the two groups of villages were calculated, and the rate of smoking cessation among the participants was examined.

### Quality control

To maximize the research quality, the local health personnel received professional training in the authors' hospital and took related examinations before they performed intervention tasks. Their rights and obligations were also explained to them in detail, and detailed assessment criteria were developed for evaluating their work. They received assessments every 6 months throughout the course of the study. If they failed an examination, they were retrained, and they were refused participation in the study if they could not do their jobs well. To encourage the local health personnel's enthusiasm, the authors provided free skills training in the affiliated hospital, along with some other types of reward.

### Statistical analysis

The SPSS 19.0 software package was adopted for statistical analysis. A normality test for distribution of the data was carried out using the Kolmogorov-Smirnov test, which showed that the data were normally distributed at P>0.05. Normally distributed measurement data are reported as means±SD, and the intervention and control groups were compared by the independent-sample *t*-test. Abnormally distributed measurement data are described as the median (P_50_) and percentiles (P_25_, P_75_). Accordingly, the Mann-Whitney test was applied to the two sets of data. Enumeration data are reported as frequency (constituent ratio). Comparison between the groups was performed by the Pearson *χ^2^* test. The differences were statistically significant if <0.05.

## Results

### Baseline and follow-up data

Of the 1008 participants who fulfilled the study criteria, 494 (454 men and 40 women [mean age, 54 ± 10 years]) were allocated to the intervention group and 514 (482 men and 32 women [mean age, 53 ± 10 years]) to the control group. The baseline characteristics of the two groups were generally well matched, although they differed in smoking indices (smoking indices = number of cigarettes smoked per day × years of smoking; the intervention group had higher smoking indices than the control group, 240 *vs* 180, <0.05) (Table 2). The majority of the participants were determined to be at high risk of COPD (89% in the intervention villages and 89% in the control villages).

**Table:**
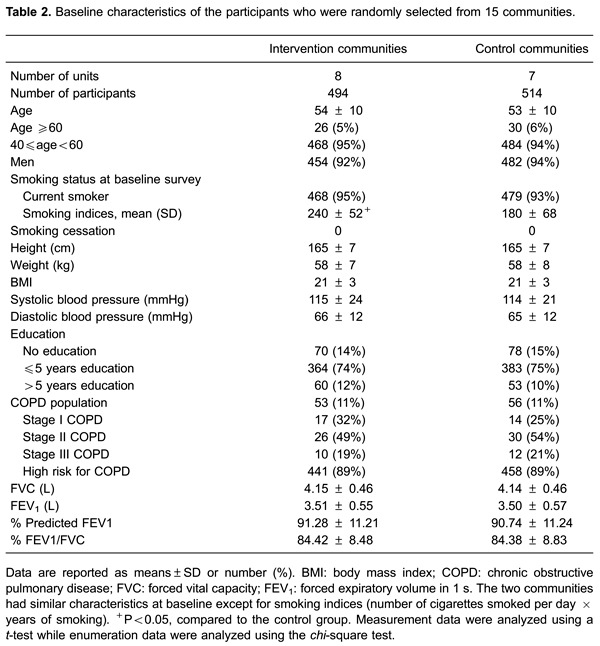

All of the patients were followed up for 18 years, or until contact was lost or they died (if this occurred before the end of the 18-year study period). In the intervention group, 78 patients died, 33 of which from COPD. In the control group, 89 patients died, 55 of which from COPD. A total of 804 (80%) participants from the baseline sample participated in the final survey (81% retention for the intervention group and 79% for the control group) (Figure 1).

### Incidence and mortality of COPD

After 18 years intervention, there were 36 new COPD patients in the intervention group and 62 in the control group. The incidence of COPD was significantly lower in the intervention group than in the control group (<0.05; Table 3). Among the patients with COPD, 33 of them died of COPD in the intervention group and 55 died of COPD in the control group. The mortality rate of COPD was significantly lower in the intervention group than in the control group (37% *vs*47%, <0.05; Table 3). However, there was no significant difference in cumulative death from all causes between the two groups (16% *vs* 17%, P>0.05; Table 3).

**Table:**
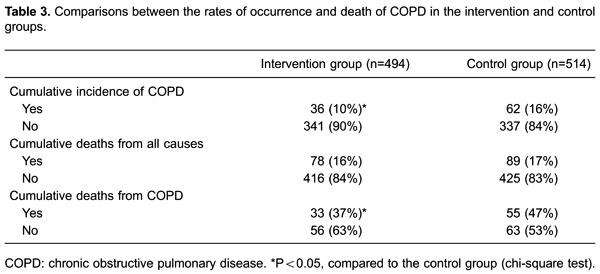

### Effect of intervention on decline of lung function

After 18 years intervention, the decline in pulmonary function in the intervention group was significantly lower than that in the control group in COPD patients and high-risk patients (Table 4). In COPD patients, FEV_1_ in the intervention group declined 470 mL, while that in the control group declined 580 mL compared with baseline values (<0.05). In participants without COPD, FEV_1_ in the intervention group declined 330 mL, while that in the control group declined 480 mL compared with baseline values (<0.01). Similarly, the decline in FVC in the intervention group was lower than that in the control group for COPD patients (390 *vs* 450 mL, <0.05) and for participants without COPD (240 *vs* 350 mL, <0.05). In COPD patients, the decline in the FEV_1_/FVC ratio in the intervention group was lower than that in the control group compared with baseline values (6% *vs*9%, <0.05). In participants without COPD, the decline in the FEV_1_/FVC ratio in the intervention group was lower than that in the control group compared with baseline values (2% *vs*4%, <0.05).

**Table:**
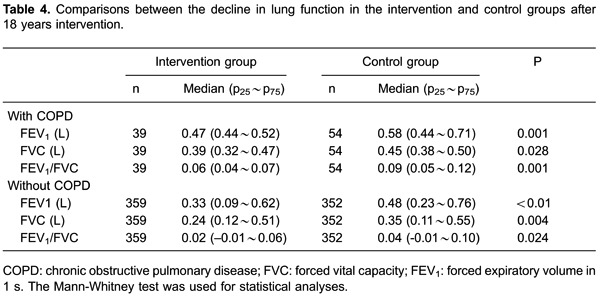

### Effect of intervention on lung function

After 18 years intervention, pulmonary function in the intervention group was better than that in the control group (Table 5). In COPD patients, mean FEV_1_ in the intervention group was higher than that in the control group (2.26 *vs* 1.89 L, <0.01). Mean FVC in the intervention group was higher than that in the control group (3.81 *vs* 3.45 L, <0.01). The mean FEV_1_/FVC ratio in the intervention group was higher than that in the control group (0.59 *vs* 0.55, <0.05).

Similar results were found for participants without COPD. Mean FEV_1_ in the intervention group was higher than that in the control group (3.21 *vs* 3.13 L, <0.05). The mean FEV_1_/FVC ratio was higher in the intervention group than that in the control group (0.84 *vs* 0.83, <0.05). However, there was no significant difference in mean FVC between the intervention and control groups (3.81 *vs* 3.78 L, P>0.05).

**Table:**
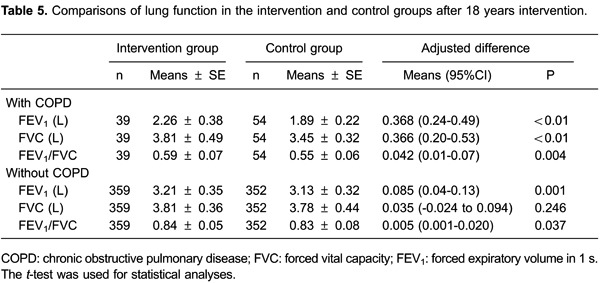

### Changes in smoking status

At baseline, there was no significant difference in smoking rate between the two groups (95% *vs* 93%, P>0.05). At the end of the study, the smoking rate in the control group was higher than that in the intervention group (79% *vs* 62%, <0.05). Additionally, the smoking indices of current smokers in the intervention group were significantly lower than those in the intervention group (450 *vs* 350, <0.05).

## Discussion

Although many studies on COPD interventions in outpatients have been conducted, they have generally been conducted in urban or economically developed areas. Patients in economically underdeveloped or rural areas have received less attention, especially in Chinese rural areas. To date, inadequate attention has been paid to conditions of COPD in Chinese rural areas. Morbidity of COPD in rural areas in China is higher than that in the city. Additionally, numerous rural COPD patients have failed to receive effective diagnosis and treatment before the disease worsens. In this case, Chinese rural residents are in great need of comprehensive intervention measures, including prevention of COPD, as well as daily disease control and treatment. To the best of our knowledge, the current study is the first randomized controlled trial to examine the effectiveness of a community-based comprehensive intervention for COPD in a rural area of China. We found that such an intervention effectively reduced the incidence of COPD among a high-risk population, delayed a decline in lung function, and improved the prognosis of COPD patients and those at risk. The current findings may inspire creation of new prevention and treatment programs for COPD for populations of poor and underdeveloped areas.

Currently, COPD is the fourth leading cause of death globally (9) and it is projected to have the fifth leading burden of disease worldwide by 2020 (10). Despite these facts, COPD fails to receive sufficient attention either from the general population or from the health care community. In the East and the West, a considerable proportion of patients do not receive the correct diagnosis of COPD, even when they already have major symptoms (11,12). The reasons for this phenomenon are multifaceted. Because of a lack of diagnostic equipment or of clinical experience, some primary care physicians and general physicians are unable to accurately identify COPD (13,14). Many patients also lack knowledge of COPD and do not request medical help, even when they have respiratory symptoms (15). This phenomenon is particularly acute in China's rural areas. China's rural areas tend to be remote, and their residents tend to lead a self-sufficient lifestyle with relatively little connection with the outside world. Most rural residents only have a primary level of education, and some of them are illiterate. Some rural residents live in poverty, and levels of education and income can affect people's health in a variety of ways. Compared with patients who have a higher education or income levels, those with low education and income levels tend to have poorer health outcomes (16–20), especially when they suffer from chronic diseases, such as chronic immunodeficiency diseases, chronic heart failure, diabetes, or COPD (21–25). In the present study, integrated intervention helped patients from poor rural areas who were either illiterate or had only a primary school education to improve their situation and the prognosis of COPD.

COPD may be promoted by a variety of external factors, such as air pollution, malnutrition, and smoking, of which smoking is the major cause. The relationship between tobacco use and COPD has already been recognized. Smoking can significantly increase an individual's likelihood of contracting COPD (26) and smoking rates tend to be high among patients with COPD (27). In the present study, almost all of those who qualified for the study were current smokers. The reason for this finding might be due to the social context of China's rural areas. These areas tend to be remote and underdeveloped, and lack entertainment and leisure facilities. Smoking is one of the main leisure activities of Chinese agricultural workers. Throughout China, friends and relatives often present cigarettes to each other as gifts.

Quitting smoking is one of the most important measures to prevent COPD and smoking cessation is also considered the most effective intervention for slowing down the disease progression of COPD. Persuasion to quit smoking is one of the most important and comprehensive treatment measures for COPD (28). Smokers with COPD tend to have higher tobacco consumption, higher dependence on nicotine, higher concentrations of CO in exhaled air, and greater difficulty quitting smoking compared with healthy smokers (29). Smoking cessation strategies include pharmacological therapy and behavioral counseling. In previous studies, pharmacological therapy together with behavioral counseling were found to be the most effective smoking cessation strategy for COPD patients, while neither counseling alone nor particular anti-smoking drugs alone effectively improve smoking cessation rates (30). However, in the current study, behavioral counseling was an effective smoking cessation measure. The local health personnel in our study might deserve most of the credit for this success, because the close connections between them and the patients might have improved patients' therapeutic adherence. In the present study, a lower incidence of COPD and a delay in decline in lung function in the intervention group compared with the control group may partly be attributed to smoking cessation, as well as simple pulmonary rehabilitation.

### Limitations of the study

This study has some limitations. First, most of the subjects in the study were male (454 men and 40 women in the intervention group; 482 men and 32 women in the control group). Although COPD has a higher incidence in men than in women in China (2), there is still a large number of female patients with COPD in China. They receive even less attention than male patients. Representative numbers of female and male patients with COPD should be included in similar studies in the future. Second, because COPD has a long-term course and entails a slow decline in lung function, regularly monitoring lung function is useful for assessing the condition of patients. However, in the present study, because of funding and other restrictions, pulmonary function testing was not conducted for all of the participants every year. Most of the patients were tested only at the beginning and end of the study, so we were unable to collect detailed information on the dynamic changes in patients' lung function. Therefore, such detailed information could not be used to guide the present research. Finally, there are some questionnaires, such as the St. George Respiratory Questionnaire, that are widely used in the study of COPD. In the present study, a questionnaire was initially used as a research tool. However, in practice, the patients appeared to have difficulty in responding to some of the questions. They often did not correctly understand the choices and made incorrect judgments. This might be related to their relatively low level of education. To ensure rigor of the study, a similar tool should be developed for use in poorly educated populations.

In summary, a rural community-based integrated intervention significantly helped decrease the COPD-associated rate of morbidity among the high-risk population by delaying a decline in lung function and improving prognoses through simple, economical, multidisciplinary co-operation between the researchers and the local medical staff. Although the results were obtained in a rural area of China, and thus might not apply to other areas, medical staff and health policy makers worldwide will hopefully find the present results useful.
